# Dual Regulation of Tank Binding Kinase 1 by BRG1 in Hepatocytes Contributes to Reactive Oxygen Species Production

**DOI:** 10.3389/fcell.2021.745985

**Published:** 2021-10-01

**Authors:** Fangqiao Lv, Tinghui Shao, Yujia Xue, Xiulian Miao, Yan Guo, Yutong Wang, Yong Xu

**Affiliations:** ^1^Department of Cell Biology, Municipal Laboratory for Liver Protection and Regulation of Regeneration, School of Basic Medical Sciences, Capital Medical University, Beijing, China; ^2^Key Laboratory of Targeted Intervention of Cardiovascular Disease and Collaborative Innovation Center for Cardiovascular Translational Medicine, Department of Pathophysiology, Nanjing Medical University, Nanjing, China; ^3^College of Life Sciences and Institute of Biomedical Research, Liaocheng University, Liaocheng, China

**Keywords:** transcription regulation, chromatin remodeling protein, kinase, phosphorylation, ROS, hepatocyte, non-alcoholic fatty liver disease

## Abstract

Excessive accumulation of reactive oxygen species (ROS) is considered a major culprit for the pathogenesis of non-alcoholic fatty liver disease (NAFLD). We have previously shown that deletion of Brahma related gene 1 (BRG1) mitigated NAFLD in mice in part by attenuating ROS production in hepatocyte. Here we report that BRG1 deletion led to simultaneous down-regulation in expression and phosphorylation of tank binding kinase 1 (TBK1) *in vivo* and *in vitro*. On the one hand, BRG1 interacted with AP-1 to bind to the TBK1 promoter and directly activated TBK1 transcription in hepatocytes. On the other hand, BRG1 interacted with Sp1 to activate the transcription of c-SRC, a tyrosine kinase essential for TBK1 phosphorylation. Over-expression of c-SRC and TBK1 corrected the deficiency in ROS production in BRG1-null hepatocytes whereas depletion of TBK1 or c-SRC attenuated ROS production. In conclusion, our data suggest that dual regulation of TBK1 activity, at the transcription level and the post-transcriptional level, by BRG1 may constitute an important mechanism underlying excessive ROS production in hepatocytes.

## Introduction

Non-alcoholic fatty liver disease (NAFLD) represents a continuum of pathologies ranging from simple steatosis, to steatohepatitis, to cirrhosis (Michelotti et al., 2013). Due to changes in life styles and dietary choices, recent decades have seen drastically increased prevalence of NAFLD, which is projected to become the most important underlying cause for hepatocellular carcinoma and liver transplantation (Anstee et al., 2019). Despite advances in the understanding of its pathogenesis, therapeutic solutions for NAFLD are limited. Several promising drugs are currently undergoing clinical trials for NAFLD treatment though definitive conclusions are yet to be reached due to the SARS-Cov2 pandemic (Abdelmalek, 2021). The mechanisms that contribute to NAFLD pathogenesis are complex, which include aberrant systemic and hepatic metabolism, low-magnitude sterile inflammation (i.e., meta-inflammation), and oxidative stress owing to excessive production and/or ineffective removal of reactive oxygen species (ROS) (Wesolowski et al., 2017).

ROS serves as a double-edged sword being an important host defense mechanism on the one hand and a disruptor of homeostasis on the other (Sies and Jones, 2020). Landmark studies have strongly implicated ROS in NAFLD pathogenesis. Major markers for oxidative stress have been detected to be present in significantly elevated levels in the liver (Weltman et al., 1998; Seki et al., 2002; Chalasani et al., 2003; Videla et al., 2004; Oliveira et al., 2005) and in the circulation (Koruk et al., 2004; Horoz et al., 2005) in patients with NAFLD. The causal relationship between ROS and NAFLD is further demonstrated by studies employing transgenic animals in which key molecules involved in ROS production/cleansing are manipulated. For instance, deletion of individual NADPH oxidase (NOX) proteins, which play key roles in catalyzing ROS generation, in mice mitigates NAFLD (Bettaieb et al., 2015; Matsumoto et al., 2018; Mukherjee et al., 2018). On the contrary, deficiency of nuclear factor erythroid 2–related factor 2 (Nrf2), a major anti-oxidative transcription factor, renders the mice more susceptible to the development of NAFLD (Chowdhry et al., 2010; Sugimoto et al., 2010; Meakin et al., 2014). In addition, several clinical studies have reported that administration of antioxidant supplements may be associated with beneficial effects in NAFLD patients although most of these studies are not considered to be conclusive (Salomone et al., 2016; Ferramosca et al., 2017).

BRG1 is a chromatin remodeling protein playing versatile regulatory roles in human pathophysiology. Recent studies have portrayed BRG1 as a critical regulator of redox signaling in a wide range of tissues and cells (You et al., 2020). Investigations conducted in our laboratory have systemically profiled the role of BRG1 in NAFLD pathogenesis. BRG1 is able to interact with nuclear factor kappa B (NF-κB) to orchestrate a transcriptional program in hepatocytes promoting the production of pro-inflammatory mediators (Tian et al., 2013). BRG1 can also interact with sterol response element binding protein (SREBP), the master regulator of lipogenesis, to enhance the synthesis of triglycerides and cholesterol in hepatocytes (Li et al., 2018; Fan et al., 2020; Kong et al., 2021). More importantly, conditional BRG1 knockout mice are protected from NAFLD partly because BRG1 can directly bind to the NOX promoter and activate NOX transcription to promote ROS production in hepatocytes (Liu et al., 2019).

Tank binding kinase 1 (TBK1), also known as NF-κB activating kinase (NAK) or TRAF2 associated kinase (T2K), was initially identified as an upstream regulator of NF-κB signaling independent of inhibitor of kappa B (IκB) (Pomerantz and Baltimore, 1999; Bonnard et al., 2000; Tojima et al., 2000). TBK1 is an atypical kinase whose activity is primarily regulated by autophosphorylation although upstream priming kinases for TBK1 have also been reported (Ma et al., 2012). Mounting evidence suggests that TBK1 is a regulator of NAFLD pathogenesis by programming the innate immune response and by altering hepatic metabolism (Reilly et al., 2013; Cruz et al., 2018; Huh et al., 2020). However, it is not clear (1) whether or not BRG1 may influence TBK1 expression/activity and (2) whether TBK1 may regulate ROS production in the context of NAFLD. Here we report that BRG1 can simultaneously activate TBK1 expression and modulate TBK1 phosphorylation in hepatocytes, which may contribute to ROS production in hepatocytes.

## Materials and Methods

### Animals

All animal experiments were reviewed and approved by the Nanjing Medical University Ethics Committee on Humane Treatment of Experimental Animals (approval #: IACUC-1811060). The mice were maintained in an SPF environment with 12 h light/dark cycles and libitum access to food and water. All animal procedures conform to the guidelines on the protection of animals per the current NIH guidelines. Hepatocyte BRG1 conditional deletion mice were generated by crossing the *Smarca4*^f/f^ mice to the *Alb*-Cre mice as previously described (Hong et al., 2020; Dong et al., 2021; Kong et al., 2021). To induce non-alcoholic fatty liver disease (NAFLD), the mice were fed a high-fat high-carbohydrate diet (D12492, Research Diets) for 16 consecutive weeks or a methionine- and choline-deficient (MCD) diet (A02082002B, Research Diets) for eight consecutive weeks as previously described (Fan et al., 2020; Kong et al., 2021). Euthanasia was performed by intraperitoneal injection with pentobarbital sodium (150 mg/kg).

### Cell Culture, Plasmids, Transient Transfection, and Reporter Assay

Primary hepatocytes were isolated from WT and BRG1^LKO^ mice and treated with palmitate (0.4 mM) for 12 h as previously described (Hong et al., 2020; Dong et al., 2021). The TBK1 promoter-luciferase constructs (Li et al., 2003) and the SRC promoter-luciferase constructs (Ritchie et al., 2000) have been described previously. Mutant constructs were generated by a QuikChange kit (Thermo Fisher, Waltham, MA, United States) and verified by direct sequencing. Small interfering RNAs purchased from Dharmacon. Transient transfection was performed with Lipofectamine 2000. Cells were harvested 48 h after transfection. Luciferase activities were assayed 24–48 h after transfection using a luciferase reporter assay system (Promega) as previously described (Chen et al., 2021; Liu et al., 2021a,b; Zhang et al., 2021). For luciferase assay/DHE staining/luminescence assay, the cells were plated at the density of ∼1 × 10^5^ cells/well in 12-well culture dishes. For RNA extraction-qPCR experiments, the cells were plated at the density of ∼2 × 10^5^ cells/well in p35 culture dishes. For protein extraction-Western blotting experiments, the cells were plated at the density of ∼5 × 10^5^ cells/well in p60 culture dishes. For ChIP assay, the cells were plated at the density of ∼2 × 10^6^ cells/well in p100 culture dishes. Palmitate was dissolved in pre-heated 0.1 N NaOH and diluted 1:10 in pre-warmed 12% BSA solution at the final stock concentration of 10 mM. Stock palmitate solution was diluted with culture media to a final concentration of 0.4 mM and added to the cells; equivalent volume of vehicles was added as a control.

### Protein Extraction and Western Blot

Whole cell lysates were obtained by re-suspending cell pellets in RIPA buffer (50 mM Tris pH7.4, 150 mM NaCl, 1% Triton X-100) with freshly added protease inhibitor (Roche) as previously described (Chen et al., 2020a; Sun et al., 2020; Wu T. et al., 2020; Wu X. et al., 2020). Western blot analyses were performed with anti-TBK1 (1:1,000, Abcam, ab40676), anti-c-Jun (1:2,000, Proteintech, 24909-1), anti-c-Fos (1:2,000, Proteintech, 66590-1), anti-Sp1 (1:1,000, Abcam, ab227383), anti-phospho-S172-TBK1 (1:1,000, Cell Signaling Tech, 5483), anti-SRC (1:1,000, Abcam, ab16885), and anti-β-actin (1:5,000, Sigma, A2228) antibodies. The anti-phospho-Y179-TBK antibody was custom-made using the synthesized peptide TEE(pY)LHPDM-C as immunogen for injection in Balb/c mice as previously described (Li et al., 2017). For densitometrical quantification, densities of target proteins were normalized to those of β-actin as previously described (Chen et al., 2020b,c; Li et al., 2020a; Mao et al., 2020a). Data are expressed as relative protein levels compared to the control group which is arbitrarily set as 1.

### RNA Isolation and Real-Time PCR

RNA was extracted with the RNeasy RNA isolation kit (Qiagen). Reverse transcriptase reactions were performed using a SuperScript First-strand Synthesis System (Invitrogen) as previously described (Dong et al., 2020; Li et al., 2020b,c; Lv et al., 2020). Real-time PCR reactions were performed on an ABI Prism 7500 system with the following primers: *Tbk1*, 5′-CTCATCACAGCCTACGGAGAC-3′ and 5′-CGTGTTGGAA TTGGTTGAGTCG-3′; *Src*, 5′-CCGAGCGGCTTCTTTACCC-3′ and 5′-GCATGATACATGATGCGGTAGT-3′. Ct values of target genes were normalized to the Ct values of housekeeping control gene (18s, 5′-CGCGGTTCTATTTTGTTGGT-3′ and 5′-TCGTCTTCGAAACTCCGACT-3′ for both human and mouse genes) using the ΔΔCt method and expressed as relative mRNA expression levels compared to the control group which is arbitrarily set as 1.

### Chromatin Immunoprecipitation

Chromatin immunoprecipitation (ChIP) assays were performed essentially as described before (Mao et al., 2020b; Yang et al., 2020a,b). In brief, chromatin in control and treated cells were cross-linked with 1% formaldehyde. Cells were incubated in lysis buffer (150 mM NaCl, 25 mM Tris pH 7.5, 1% Triton X-100, 0.1% SDS, 0.5% deoxycholate) supplemented with protease inhibitor tablet and PMSF. DNA was fragmented into ∼200 bp pieces using a Branson 250 sonicator. Aliquots of lysates containing 200 μg of protein were used for each immunoprecipitation reaction with anti-BRG1 (5 μg/reaction, Santa Cruz, sc-10768) or pre-immune IgG. Precipitated genomic DNA was amplified by real-time PCR. A total of 10% of the starting material is also included as the input. Data are then normalized to the input and expressed as% recovery relative the input as previously described (Chen et al., 2020b,c). All experiments were performed in triplicate wells and repeated three times.

### Dihydroethidium Staining

Dihydroethidium (DHE) staining was performed essentially as previously described (Coarfa et al., 2020; Maity et al., 2020; Wang J. N. et al., 2020; Wang S. et al., 2020; Marti et al., 2021). Primary hepatocytes were stained with DHE (10 μM) at 37°C for 30 min. Fluorescence was visualized by con-focal microscopy (LSM 710, Zeiss) using an excitation wavelength between 480 and 520 nm and an emission wavelength between 570 and 600 nm. Quantifications were performed with Image J.

### Luminescence Reactive Oxygen Species Assay

Quantitative measurements of intracellular ROS were performed with a ROS-Glo system (G8820, Promega). Briefly, a luminescence substrate solution was added to and incubated with cultured cells for 6 h followed by the addition of the diction solution. Luminescence was measured using a GloMax microplate reader (GM3000, Promega). Data were expressed as relative ROS levels compared to the control group.

### Statistical Analysis

Sample sizes reflected the minimal number needed for statistical significance based on power analysis and prior experience. Sample size for all animal experiments was ≥4 while all *in vitro* experiments were repeated at least three times with a power analysis showing β > 80. Data are expressed as mean ± standard deviation (SD). For comparison between two groups, two-tailed, unpaired Student’s *t*-test was performed. For comparison between more than two groups, one-way ANOVA with *post hoc* Scheffe analyses were performed using an SPSS package. Unless otherwise specified, *p* values smaller than 0.05 were considered statistically significant.

## Results

### BRG1 Deletion Down-Regulates TBK1 Expression

**FIGURE 1:**
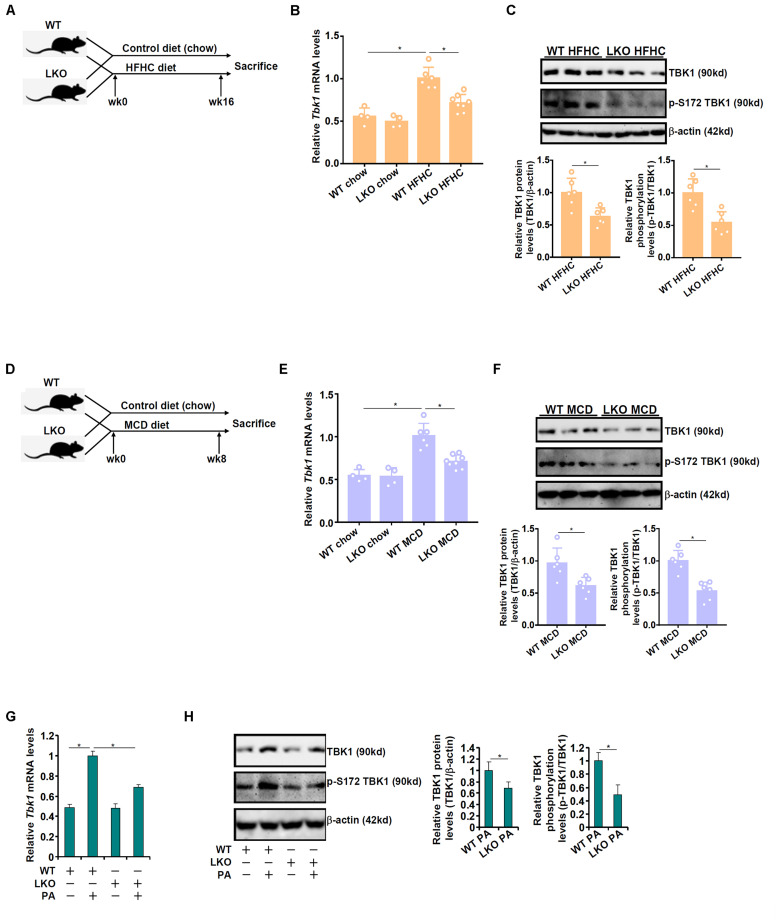
BRG1 deletion down-regulates TBK1 expression. **(A–C)** WT and BRG1^LKO^ mice were fed an HFHC diet or a chow diet for 16 weeks. **(A)** A flow chart of animal protocol. TBK1 expression in the liver was examined by qPCR **(B)** and Western **(C)**. Each lane represents a sample extracted from an individual mouse. *N* = 4 mice for the chow groups and *N* = 6 mice for the HFHC groups. *p < 0.05. (one-way ANOVA with *post hoc* Scheffe test for qPCR quantification and two-tailed student’s *t*-test for Western quantification). **(D–F)** WT and BRG1^LKO^ mice were fed an MCD diet or a chow diet for 8 weeks. **(D)** A flow chart of animal protocol. TBK1 expression in the liver was examined by qPCR **(E)** and Western **(F)**. Each lane represents a sample extracted from an individual mouse. *N* = 4 mice for the chow groups and *N* = 6 mice for the MCD groups. *p < 0.05 (one-way ANOVA with *post hoc* Scheffe test for qPCR quantification and two-tailed student’s *t*-test for Western quantification). **(G,H)** Primary hepatocytes were isolated from WT and BRG1^LKO^ mice and treated with palmitate (PA, 0.4 mM) for 12 h. TBK1 expression was examined by qPCR and Western. *p < 0.05 (one-way ANOVA with *post hoc* Scheffe test). Data are expressed as mean ± standard deviation (SD). All experiments were performed in triplicate wells and repeated three times. One representative experiment is shown.

The effect of BRG1 deficiency on TBK1 expression was examined by the following experiments. Liver conditional BRG1 knockout (BRG1^LKO^) mice and wild type (WT) littermates were fed a high-fat high carbohydrate (HFHC) diet for 16 weeks to induce non-alcoholic fatty liver disease (Figure 1A). As shown in Figure 1B, TBK1 mRNA expression was significantly up-regulated in the HFHC-fed livers compared to the chow diet-fed livers; BRG1 deletion, however, dampened in the induction of TBK1 expression by 30%. Western blotting showed that TBK1 protein levels were similarly down-regulated in the HFHC-fed LKO livers compared to the HFHC-fed WT livers (Figure 1C). Of note, TBK1 expression, at both mRNA (Figure 1B) and protein (data not shown) levels, was comparable between the chow-fed WT livers and the chow-fed LKO livers. In the second animal model, the BRG1^LKO^ mice and the WT mice were fed a methionine-and-choline deficient (MCD) diet for 8 weeks (Figure 1D). TBK1 mRNA expression was up-regulated by 1.88x fold in the WT livers but only 1.33x fold in the LKO livers equivalent to 29% suppression (Figure 1E). Similar observations were made with regard to the TBK1 protein expression (Figure 1F). Primary hepatocytes were isolated from WT and LKO mice and treated with palmitate (PA). PA treatment induced TBK1 expression more strongly in the WT cells than in the LKO cells (Figures 1G,H). Together, these data suggest that BRG1 may regulate TBK1 expression in the context of NAFLD.

### BRG1 Interacts With AP-1 to Activate TBK1 Transcription

**FIGURE 2:**
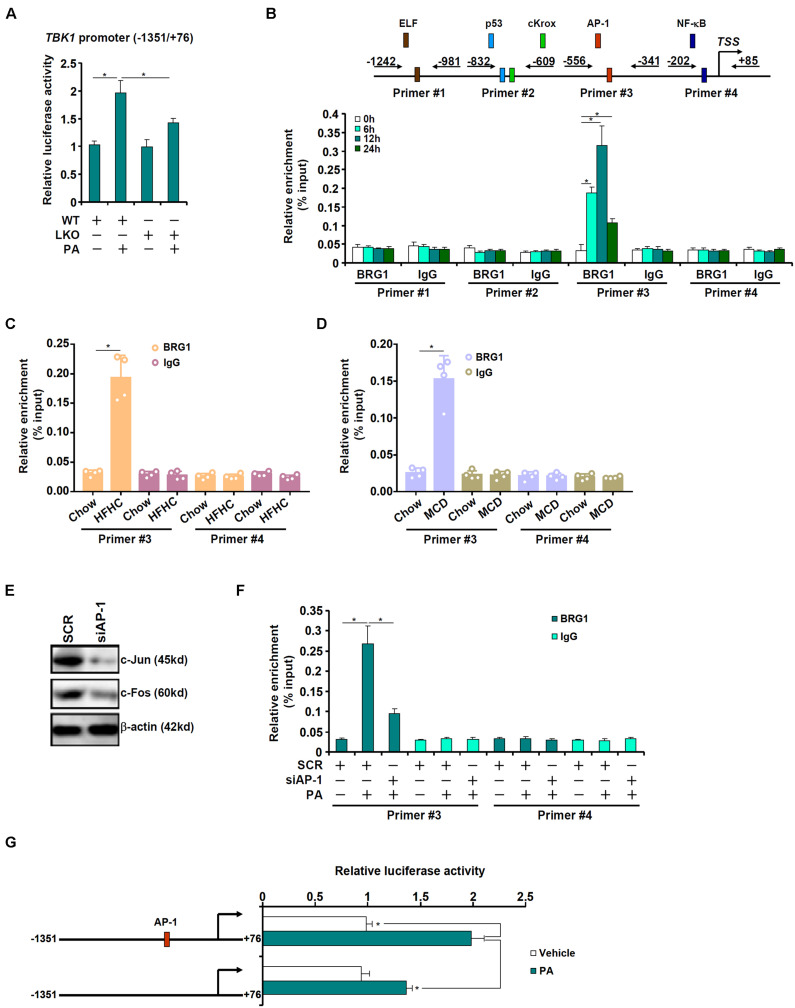
BRG1 interacts with AP-1 to activate TBK1 transcription. **(A)** A TBK1 promoter-luciferase construct (–1,351/ + 76) was transfected into primary hepatocytes isolated from WT and BRG1^LKO^ mice followed by treatment with PA (0.4 mM). Luciferase activities were normalized by protein concentration and GFP fluorescence. **(B)** Primary hepatocytes were treated with PA (0.4 mM) and harvested at indicated time points. ChIP assay was performed with anti-BRG1 or IgG. **(C)** C57B6/L mice were fed an HFHC diet for 16 weeks. ChIP assay was performed with anti-BRG1 or IgG. **(D)** C57B6/L mice were fed an MCD diet for 8 weeks. ChIP assay was performed with anti-BRG1 or IgG. **(E,F)** Primary hepatocytes were transfected with indicated siRNAs followed by treatment with PA (0.4 mM) for 12 h. Knockdown efficiency was verified by Western blotting. ChIP assay was performed with anti-BRG1. **(G)** Wild type or mutant TBK1 promoter-luciferase construct was transfected into primary hepatocytes followed by treatment with PA (0.4 mM). Luciferase activities were normalized by protein concentration and GFP fluorescence. *p < 0.05 (one-way ANOVA with *post hoc* Scheffe test). Data are expressed as mean ± standard deviation (SD). All experiments were performed in triplicate wells and repeated three times. One representative experiment is shown.

The next series of experiments were performed to evaluate the mechanism whereby BRG1 may regulate TBK1 expression. A TBK1 promoter-luciferase construct was transfected into primary hepatocytes isolated from WT and LKO mice. As shown in Figure 2A, PA treatment augmented the TBK1 promoter activity in the WT hepatocytes but barely so in the LKO hepatocytes, suggesting that BRG1 may directly regulate TBK1 transcription. Next, chromatin immunoprecipitation (ChIP) was performed to determine the region of the TBK1 promoter BRG1 may bind to. Precipitated DNA was amplified by four pairs of primers spanning the ∼1.2 kb region of the proximal TBK1 promoter: primers #1 amplified a region containing a putative E74-like factor (ELF) motif, primers #2 amplified a region containing a p53 motif and a cKrox motif, primers 3 amplified a region containing an activator protein 1 (AP-1) site, and primers #4 amplified a region with an NF-κB site; PA treatment induced strong binding of BRG1 to region #3 but not other regions of the TBK1 promoter (Figure 2B). Similarly, HFHC diet feeding (Figure 2C) or MCD diet feeding (Figure 2D) enhanced the association of BRG1 with region #3 of the TBK1 promoter in the murine livers. In order to verify whether or not AP-1 might indeed be necessary for recruiting BRG1 to the TBK1 promoter, endogenous AP-1, a heterodimer of c-Jun and c-Fos, was depleted with siRNAs (Figure 2E). AP-1 knockdown significantly dampened the binding of BRG1 to the TBK1 promoter (Figure 2F). Furthermore, mutation of the AP-1 site on the TBK1 promoter abrogated the induction of promoter activity by PA treatment (Figure 2G). Collectively, these data suggest that BRG1, possibly by interacting with AP-1, may directly activate TBK1 transcription.

### BRG1 Deficiency Down-Regulates TBK1 Phosphorylation *via* c-SRC

**FIGURE 3:**
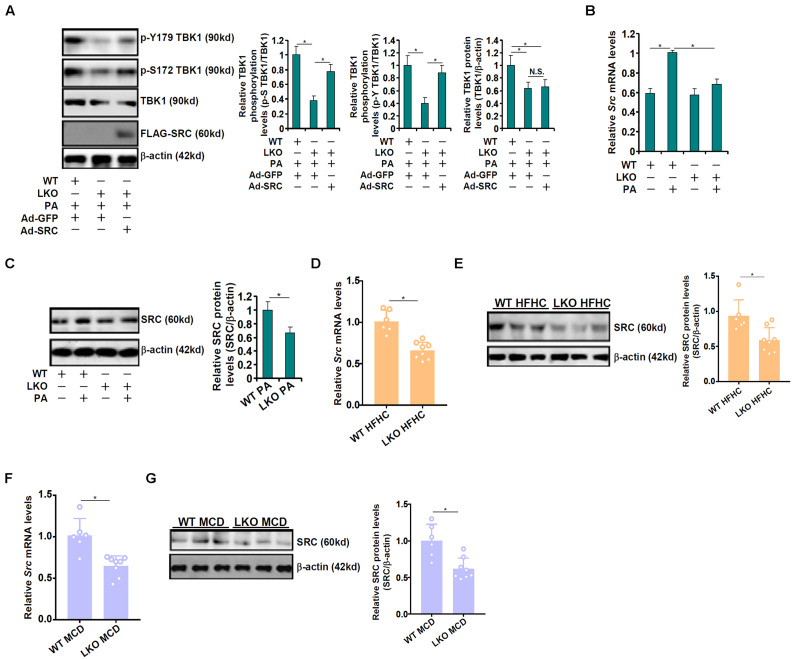
BRG1 deficiency down-regulates TBK1 phosphorylation *via* c-SRC. **(A)** Primary hepatocytes isolated from WT and BRG1^LKO^ mice were transduced with indicated adenovirus followed by treatment with PA (0.4 mM). TBK1 phosphorylation was examined by Western. **(B,C)** WT and BRG1^LKO^ mice were fed an HFHC diet or a chow diet for 16 weeks. SRC expression in the liver was examined by qPCR and Western. Each lane represents a sample extracted from an individual mouse. *p < 0.05 (one-way ANOVA with *post hoc* Scheffe test for qPCR quantification and two-tailed student’s *t*-test for Western quantification). **(D,E)** WT and BRG1^LKO^ mice were fed an MCD diet or a chow diet for 8 weeks. SRC expression in the liver was examined by qPCR and Western. Each lane represents a sample extracted from an individual mouse. *p < 0.05 (one-way ANOVA with *post hoc* Scheffe test for qPCR quantification and two-tailed student’s *t*-test for Western quantification). **(F,G)** Primary hepatocytes were isolated from WT and BRG1^LKO^ mice and treated with palmitate (PA, 0.4 mM) for 12 h. SRC expression was examined by qPCR and Western. *p < 0.05 (one-way ANOVA with *post hoc* Scheffe test). Data are expressed as mean ± standard deviation (SD). All experiments were performed in triplicate wells and repeated three times. One representative experiment is shown.

Western blotting data showed that BRG1 deficiency, in addition to causing a decrease in total TBK1 expression, also led to suppression of TBK1 activity as indicated by reduced serine 172 (S172) phosphorylation (Figures 1C,F,H). Of note, tyrosine 179 (Y179) phosphorylation of TBK1 was similarly suppressed by BRG1 deficiency (Figure 3A). Li et al. (2017) have previously reported that c-SRC mediated Y179 phosphorylation of TBK1 serves as a prerequisite for S172 phosphorylation and hence TBK1 activation. Of interest, over-expression of c-SRC by adenovirus transduction normalized both Y179 phosphorylation and S172 phosphorylation of TBK1 in the BRG1-null hepatocytes (Figure 3A). We therefore hypothesized that BRG1 could potentially regulate SRC expression to influence TBK1 phosphorylation. QPCR (Figure 3B) and Western blotting (Figure 3C) showed that BRG1 deficiency resulted in a reduction of SRC expression in primary hepatocytes. In addition, BRG1 deletion in hepatocytes down-regulated SRC expression in the livers in the HFHC model (Figures 3D,E) and in the MCD model (Figures 3F,G).

### BRG1 Interacts With Sp1 to Activate SRC Transcription

**FIGURE 4:**
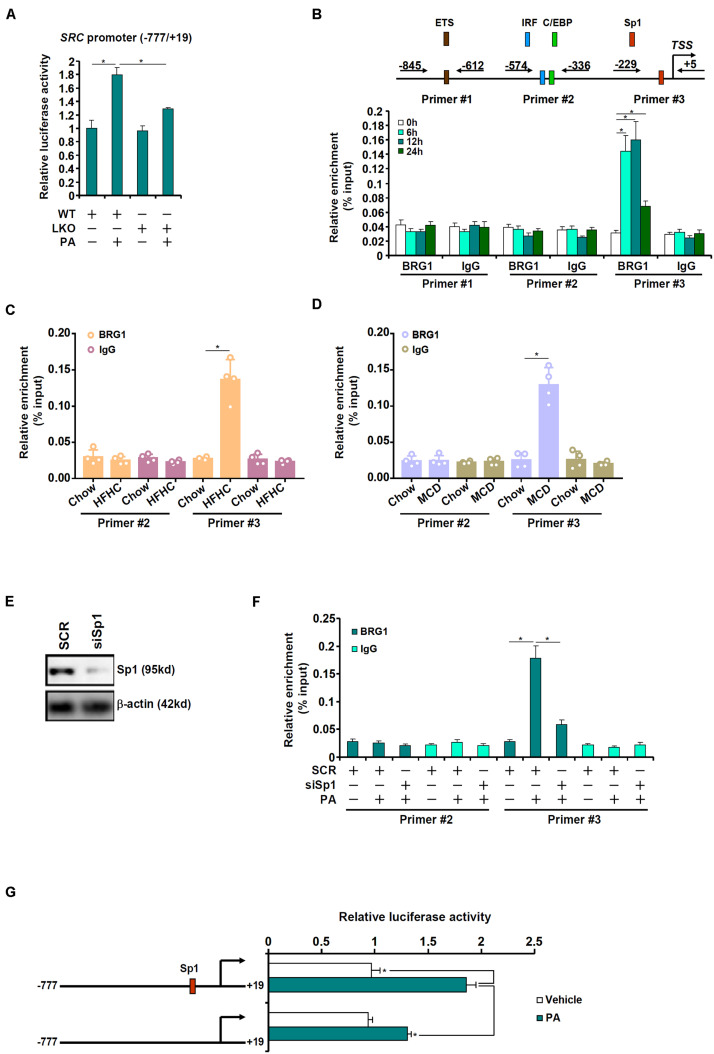
BRG1 interacts with Sp1 to activate SRC transcription. **(A)** A SRC promoter-luciferase construct (–777/ + 19) was transfected into primary hepatocytes isolated from WT and BRG1^LKO^ mice followed by treatment with PA (0.4 mM). Luciferase activities were normalized by protein concentration and GFP fluorescence. **(B)** Primary hepatocytes were treated with PA (0.4 mM) and harvested at indicated time points. ChIP assay was performed with anti-BRG1 or IgG. **(C)** C57B6/L mice were fed an HFHC diet for 16 weeks. ChIP assay was performed with anti-BRG1 or IgG. **(D)** C57B6/L mice were fed an MCD diet for 8 weeks. ChIP assay was performed with anti-BRG1 or IgG. **(E,F)** Primary hepatocytes were transfected with indicated siRNAs followed by treatment with PA (0.4 mM) for 12 h. ChIP assay was performed with anti-BRG1. **(G)** Wild type or mutant SRC promoter-luciferase construct was transfected into primary hepatocytes followed by treatment with PA (0.4 mM). Luciferase activities were normalized by protein concentration and GFP fluorescence. *p < 0.05 (one-way ANOVA with *post hoc* Scheffe test). Data are expressed as mean ± standard deviation (SD). All experiments were performed in triplicate wells and repeated three times. One representative experiment is shown.

The next set of experiments was designed to determine whether BRG1 could directly regulate SRC transcription. When a SRC promoter-luciferase construct was transfected into primary hepatocytes from WT and LKO mice, it was found that PA treatment led to more prominent induction of the SRC promoter in the WT hepatocytes than in the LKO hepatocytes (Figure 4A). Next, ChIP assay was performed in primary hepatocytes with or without PA treatment to pinpoint the region of the SRC promoter to which BRG1 might bind. As shown in Figure 4B, the proximal SRC promoter was divided into three regions and amplified by three sets of primers: primers #1 amplified the region containing a putative E26 transformation-specific (ETS) motif, primers #2 amplified the region with an interferon regulatory factor (IRF) motif and a CCAAT-enhancer binding protein (C/EBP) motif, whereas primers #3 amplified the region with a putative specificity protein 1 (Sp1) motif; PA treatment elicited strong binding of BRG1 to region #3 of the SRC promoter. ChIP assays performed with liver lysates from the HFHC diet-fed mice (Figure 4C) and the MCD diet-fed mice (Figure 4D) confirmed that BRG1 could be recruited to the SRC promoter region with an Sp1 motif. In order to authenticate the essentiality of Sp1 in BRG1 recruitment, endogenous Sp1 expression was depleted with siRNAs (Figure 4E). Sp1 depletion weakened significantly the binding of BRG1 to the SRC promoter (Figure 4F). Finally, mutation of this putative Sp1 motif within the SRC promoter canceled the induction of the promoter activity by PA treatment (Figure 4G).

### TBK1 and c-SRC Contribute to Palmitate Induced Reactive Oxygen Species Production in Hepatocytes

**FIGURE 5:**
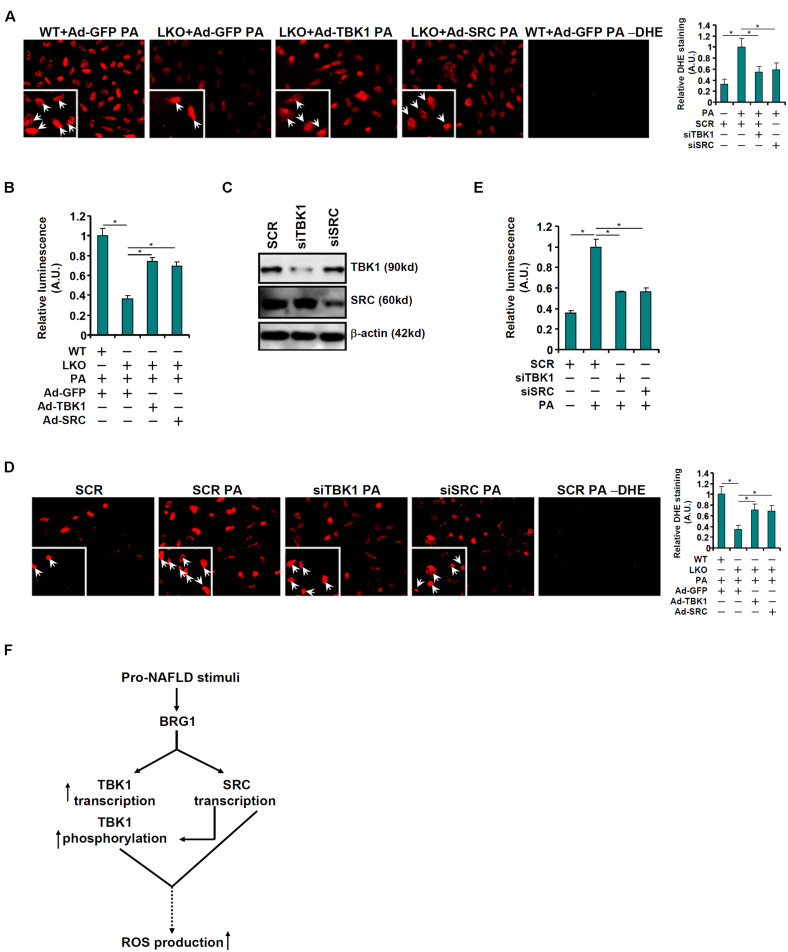
TBK1 and c-SRC contribute to PA induced ROS production in hepatocytes. **(A,B)** Primary hepatocytes isolated from WT and BRG1^LKO^ mice were transduced with indicated adenovirus followed by treatment with PA (0.4 mM). ROS levels were measured by DHE staining and luminescence assay. **(C–E)** Primary hepatocytes were transduced with indicated siRNAs followed by treatment with PA (0.4 mM). Knockdown efficiencies were examined by Western. ROS levels were measured by DHE staining and luminescence assay. *p < 0.05 (one-way ANOVA with *post hoc* Scheffe test). Data are expressed as mean ± standard deviation (SD). All experiments were performed in triplicate wells and repeated three times. One representative experiment is shown. **(F)** A schematic model.

Finally, attempts were made to correlate the observed regulation of TBK1 expression and activity by BRG1 to a functional readout relevant in the pathogenesis of non-alcoholic fatty liver disease. DHE staining showed that re-introduction of TBK1 or c-SRC *via* adenoviral delivery of ectopic expression vectors largely normalized ROS production in the BRG1-null hepatocytes bringing it to a level closer to the WT hepatocytes (Figure 5A). The restoration of ROS production by exogenous TBK1 or c-SRC over-expression in BRG1-null hepatocytes was confirmed by luminescence measurements of ROS (Figure 5B). On the contrary, knockdown of either TBK1 or c-SRC (Figure 5C) mitigated induction of ROS production by PA treatment in WT hepatocytes as measured by DHE staining (Figure 5D) and luminescence assay (Figure 5E).

## Discussion

Recent studies have clearly established the causality of TBK1 in the pathogenesis of NAFLD (Reilly et al., 2013; Cruz et al., 2018; Huh et al., 2020) although the regulation of its expression and/or activity is not completely understood. Here we provide evidence to show that BRG1 plays dual roles in the regulation of TBK1 expression/activity (Figure 5F). BRG1, *via* interacting with AP-1, directly binds to the TBK1 promoter and activates TBK1 transcription. This observation echoes our previous findings that BRG1 is an important binding partner and co-factor for AP-1, which may contribute to the transcriptional regulation of endothelin (Weng et al., 2015), galectin-3 (Li et al., 2019b), NOX4 (Li et al., 2019a), and colony stimulating factor 1 (CSF1) (Shao et al., 2019). It is noteworthy that each of these target genes for the BRG1-AP-1 complex has a causal role in NAFLD. Like BRG1, AP-1 expression and/or activity has been observed to increase in the livers in human NAFLD patients and in model animals (Dorn et al., 2014; Schulien et al., 2019). Consistently, hepatocyte-specific deletion of AP-1 retards the development of NAFLD in mice (Hasenfuss et al., 2014). Therefore, our data allude to a model in which an NAFLD-inducible transcriptional complex composed of AP-1 and BRG1 activates the transcription of multiple downstream target genes to orchestrate NAFLD pathogenesis.

TBK1 activity relies on phosphorylation of serine 172 located to the classical kinase activation loop. Although this process is considered to be auto-regulated, several distinct priming kinases have been identified. We show here that BRG1 can influence TBK1 activity indirectly by regulate the transcription of SRC, which phosphorylates TBK1 at tyrosine 179 to promote serine 172 phosphorylation (Li et al., 2017). Despite this piece of evidence, several key issues remain to be resolved. First, alternative mechanisms could account for the observation that BRG1 deficiency diminishes TBK1 phosphorylation. For instance, the calcium binding protein TBC1D9 is required for TBK1 phosphorylation by bridging increased intracellular calcium upon pathogen invasion to innate immune response (Nozawa et al., 2020). Interestingly, a recent report by Song et al. (2014) indicates that TBC1D9 might be a direct transcriptional target for BRG1. In addition, BRG1 has a role in handling calcium influx/efflux (Qiu and Ghosh, 2008; Nasipak et al., 2015; Witwicka et al., 2019; Zhu et al., 2021). Thus, considering the intimate relationship between calcium overload and ROS production (Gorlach et al., 2015), an intriguing hypothesis would be that BRG1 senses intracellular calcium fluctuation and activates TBC1D9 transcription to alter TBK1 phosphorylation. Second, although SRC expression appears to be responsive to pro-NAFLD stimuli, its implication in NAFLD pathogenesis has not been clearly defined. A recent study by Elsayed et al. (2021) has found that administration of a SRC specific inhibitor, Dasatinib, can effectively prevent the progression of NAFLD in mice fed a Western diet (WD) for 16 weeks. Dasatinib administration attenuated hepatic lipid accumulation, inflammation, and fibrosis in the WD-fed mice although it is not clear whether TBK1 activity or ROS production was altered. Global c-SRC knockout mice display osteopetrosis owing to defective osteoclast activity but the phenotype of these mice in NAFLD models has not yet been investigated (Soriano et al., 1991). Third, it has been recently reported that members of the SRC family of tyrosine kinases other than c-SRC are capable of phosphorylating TBK1 but with the opposite consequence. According to Liu et al. (2017), phosphorylation of TBK1 Y354/394 by Lck, Hck, and Fgr disrupts TBK1 dimerization and dampens TBK1 activity. Whether the antagonism between different SRC kinases with regard to TBK1 activity is functionally relevant in NAFLD pathogenesis awaits further investigation.

Our data indicate that over-expression of TBK1 or SRC rescues defective ROS production in BRG1-null cells whereas knockdown of TBK1 or SRC suppresses PA-induced ROS production in WT cells. Because TBK1 is considered a master regulator of innate immunity to fend off invading pathogens, it is easy to perceive that increased ROS production may be a part of the host defense mechanism triggered by TBK1 activation although the precise mechanism whereby TBK1 incises ROS generation is not clear at this point (Chen et al., 2018). Several well-characterized TBK1 substrates, including NF-κB, IRF3, and signal transducer and transcription activator 6 (STAT6), have been shown to promote ROS production in various cell types (Helgason et al., 2013). In addition, Nakhaei et al. (2012) have demonstrated that phosphorylation of X-Linked inhibitor of apoptosis (XIAP) by TBK1 targets XIAP to proteasomal degradation. Since XIAP is known to suppress intracellular ROS levels by activating the expression of antioxidant genes, it is reasonable to speculate that TBK1 may contribute to ROS elevation by down-regulating XIAP levels. Alternatively, aberrant ROS generation is usually considered as a result of impaired mitochondrial integrity (Zorov et al., 2014). A recent study points out that TBK1 directly interacts with and phosphorylates adenosine monophosphate activated kinase (AMPK) to inhibit AMPK activity (Zhao et al., 2018). Because a plethora of investigations have confirmed the essential role of AMPK in guarding mitochondrial homeostasis (Herzig and Shaw, 2018), we propose that the observed increase in ROS production as a result of TBK1 activation may be accounted for by AMPK inhibition. These interesting possibilities certainly deserve further attention in future studies.

Despite the advances our data provided with regard to the regulation of TBK1 by BRG1, there are several major limitations. First, the MCD diet model used in this study (Figures 1D–F) is generally considered as imperfect and does not faithfully recapitulate the pathogenesis of NAFLD in humans (Farrell et al., 2019). The data originated from this model need to be further authenticated in human NAFLD-relevant models and, ideally, in human NAFLD specimens. Second, we focused on ROS production as a functional readout for the newly identified BRG1-TBK1 axis. Multiple pathophysiological processes, including skewed glucose and lipid metabolism, altered intrahepatic immune response, accelerated fibrogenesis, and suppressed autophagy flux, are observed in and thought to collectively drive the development and progression of NAFLD (Marchesini et al., 2016). Whether the BRG1-TBK1 axis is operative in the regulation of these processes should be carefully examined in future studies.

In summary, our data illustrate a novel mechanism where transcriptional and post-transcriptional regulation of TBK1 in hepatocytes by BRG1 may contribute to aberrant ROS production. Additional studies are needed to cement the proposed model (Figure 5F) so that effective and safe therapeutic strategies can be devised for the intervention of NAFLD in humans.

## Data Availability Statement

The original contributions presented in the study are included in the article, further inquiries can be directed to the corresponding author/s.

## Ethics Statement

The animal study was reviewed and approved by Nanjing Medical University Ethics Committee on Humane Treatment of Experimental Animals.

## Author Contributions

YW and YoX conceived the project. FL, TS, YuX, XM, and YG designed experiments, performed experiments, collected data, and analyzed data. YoX wrote the manuscript. YW and FL provided funding and supervision. All authors contributed to the article and approved the submitted version.

## Conflict of Interest

The authors declare that the research was conducted in the absence of any commercial or financial relationships that could be construed as a potential conflict of interest.

## Publisher’s Note

All claims expressed in this article are solely those of the authors and do not necessarily represent those of their affiliated organizations, or those of the publisher, the editors and the reviewers. Any product that may be evaluated in this article, or claim that may be made by its manufacturer, is not guaranteed or endorsed by the publisher.
